# Designing magnetic microcapsules for cultivation and differentiation of stem cell spheroids

**DOI:** 10.1038/s41378-024-00747-9

**Published:** 2024-09-12

**Authors:** Kihak Gwon, Ether Dharmesh, Kianna M. Nguyen, Anna Marie R. Schornack, Jose M. de Hoyos-Vega, Hakan Ceylan, Gulnaz Stybayeva, Quinn P. Peterson, Alexander Revzin

**Affiliations:** 1 Department of Physiology and Biomedical Engineering, Mayo Clinic, Rochester, MN USA; 2 Department of Biofibers and Biomaterials Science, Kyungpook National University, Daegu, Republic of Korea; 3 Department of Biomedical Engineering, Saint Louis University, St. Louis, MO USA; 4 Department of Physiology and Biomedical Engineering, Mayo Clinic, Scottsdale, AZ USA

**Keywords:** Nanoparticles, Microfluidics

## Abstract

Human pluripotent stem cells (hPSCs) represent an excellent cell source for regenerative medicine and tissue engineering applications. However, there remains a need for robust and scalable differentiation of stem cells into functional adult tissues. In this paper, we sought to address this challenge by developing magnetic microcapsules carrying hPSC spheroids. A co-axial flow-focusing microfluidic device was employed to encapsulate stem cells in core-shell microcapsules that also contained iron oxide magnetic nanoparticles (MNPs). These microcapsules exhibited excellent response to an external magnetic field and could be held at a specific location. As a demonstration of utility, magnetic microcapsules were used for differentiating hPSC spheroids as suspension cultures in a stirred bioreactor. Compared to standard suspension cultures, magnetic microcapsules allowed for more efficient media change and produced improved differentiation outcomes. In the future, magnetic microcapsules may enable better and more scalable differentiation of hPSCs into adult cell types and may offer benefits for cell transplantation.

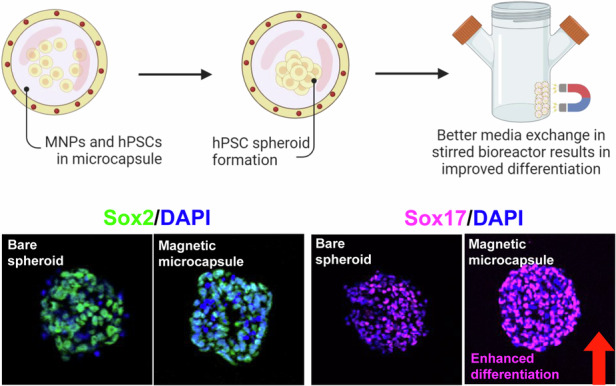

## Introduction

Human pluripotent stem cells (hPSCs) are capable of unlimited self-renewal and differentiation into all adult tissue types. These cells offer considerable promise for tissue engineering and regenerative medicine applications^1–3^. However, laboratory-scale cultures of hPSCs are typically inadequate to meet the demands of therapeutic applications, which require large quantities of cells^4–7^. Suspension cultures in spinner flasks (or stirred bioreactors) have emerged as a promising solution for scalable production of stem cells^8,9^. These bioreactors create a dynamic and tightly regulated cell environment with tunable pH, temperature, and nutrient supply to closely mimic the in vivo conditions required for optimal stem cell expansion and differentiation^9–12^. However, several issues persist in bioreactor-based cell cultures, including nutrient delivery, damage due to high levels of shear stress, and significant loss of cells during culture processes^9,13^. In particular, the use of impellers within bioreactors applies varying levels of shear stress on cells, with high shear near the tips that decreases towards the center^13^. These shear forces can alter differentiation outcomes of stem cells and lower cell viability, and need to be mitigated with countermeasures^14–16^.

Biomaterials may offer solutions to multiple challenges associated with scalable cultivation of stem cells including better protection against mechanical damage and improved differentiation^17^. Natural and synthetic biomaterials are commonly used as scaffolds in tissue and regenerative engineering, with tunable mechanical properties and the ability to incorporate soluble factors that support desired cellular functions^18–20^. Several studies have sought to utilize biomaterials to address challenges associated with bioreactor-based cell culture. Microcarriers, microbeads composed of materials such as collagen or dextran, have been employed to serve as a surface for cell adhesion within suspension cultures, offering benefits of preventing cell aggregation and increasing surface area for cell culture expansion^21–23^. However, as the stem cells are on the surface of microcarriers, they continue to experience the effects of shear stress during stirring.

Microencapsulation provides a physical barrier for cells from the shear stresses experienced in a bioreactor. Importantly, encapsulating pluripotent stem cells within biomaterial capsules continues to allow for diffusion of factors and nutrients that promote stem cell growth and differentiation^24–26^. This technique has been shown to provide protection against mechanical forces and immune response and to improve cell differentiation^27–29^. The use of biomaterials within these microencapsulation structures affords opportunity for the incoporation of bioactive components that can be utilized to enhance cellular function^30^. Microencapsulation provides additional advantages when looking ahead at the potential use of stem cell-differentiated products as cellular therapy. Microencapsulated cells are immunoisolated, preventing destruction by the host immune system while avoiding immunosuppression of the patient and its sequelae such as suceptability to life-threatening illness^31,32^. A number of strategies have been reported for fabricating microcapsules, including coaxial electrospray, electrohydrodynamic co-jetting, micro-molding, photolithography, flow lithography microfluidics, soft lithography-based imprinting, and droplet microfluidics^33–36^. Our lab has employed droplet microfluidics to fabricate microcapsules with a hydrogel shell and aqueous core^37^. These microcapsules promoted aggregation of single cells into spheroids and protected cells from shear stress associated with stirring in a bioreactor^38,39^.

Efficient media change is another challenge associated with suspension cultures in stirred bioreactors. In the process of differentiation, pluripotent stem cells proceed to through multiple development stages, each stage associated with media containing specific growth factors and small molecules. A common method of medium change in stirred bioreactors is to pause agitation, allowing cells and aggregates to settle at the bottom prior to collection of medium. In order to prevent significant cell loss, some spent medium ( ~ 20% by volume) is left in the bioreactor and may adversely affect subsequent differentiation stages^5^.

The aim of this study was to combine magnetic iron oxide nanoparticles (MNPs), with our existing droplet microfluidic system to generate externally manipulatable microcapsules. We reasoned that such microcapsules may contain hPSCs spheroids and may be remotely controlled to improve the efficiency of media changes in a stirred bioreactor. Magnetic actuation is widely used for remote microdevice manipulation and control^40,41^. The application of magnetic actuation in regenerative medicine is a growing area of interest^42^. Magnetized stem cells and spheroids have also been developed to direct migration to target sites using an external magnetic field^43–46^. Furthermore, MNPs have been used to exert mechanical forces on stem cells to induce differentiation^47,48^. To our knowledge, the incorporation of MNPs into stem cell-containing microcapsules has not been reported.

In this paper, we describe microcapsules that incorporate MNPs with HUES-8 cells (an hPSC line). We demonstrate that the presence of MNPs allows manipulation of microcapsules by an external magnetic field and does not adversely affect viability, spheroid formation, and proliferation of encapsulated stem cells. In fact, the use of magnetic microcapsules allowed for more efficient media change in a stirred bioreactor, resulting in better differentiation outcomes. Magnetic microcapsules may be used in the future to enhance scale-up of stem cell differentiation cultures and may enable transplantation of cellular therapies.

## Materials and methods

### Reagents

Four-arm polyethylene glycol (PEG) maleimide (10 kDa, PEG4MAL) was purchased from Laysan Inc. (Arab, AL, USA). PEG diacrylamide (10 kDa, PEG-DAA) was purchased from Creative PEG Works (Chapel Hill, NC, USA). Dithiothreitol (DTT), pure PEG (35 kDa), triethanolamine (TEA), mineral oil, span-80, FITC-labeled dextran (70 kDa), Triton™ X-100, bovine serum albumin (BSA), and accutase cell detachment solution were purchased from Sigma-Aldrich (St. Louis, MO, USA). Fluorescent labeled MNPs (150 nm for yellow and 350 nm for red) or control MNPs without fluorescent label (350 nm) were obtained from Spherotech (Lake Forest, IL, USA). Polydimethylsiloxane (PDMS) elastomer base and curing agent kit were purchased from Dow Corning (Ringwood, IL, USA). Neodymium magnets (rectangle type: 1” long × 1/2” wide × 1/8” thick and disk type: 1/2” diameter and 1/8” thick, magnet grade: N52 (1.5 Tesla, (T))) were purchased from McMaster-Carr (Elmhurst, IL, USA). Phosphate-buffered saline (PBS) was purchased from Corning (Manassas, NY, USA). Disposable 5 mL Bioreactor was purchased from REPROCELL (Beltsville, MD, USA). Activin A growth factor and mouse anti-Sox2 antibodies were purchased from R&D Systems (Minneapolis, MN, USA). CHIR99021 was purchased from Stemgent (Cambridge, MA, USA). Optiprep densifier, ROCK Inhibitor (Y27632), and mTeSR media were purchased from STEMCELL Technologies (Vancouver, Canada). Alexa Four 488 Phalloidin and MCDB131 medium were obtained from Thermo Scientific Inc. (Rockford, IL, USA). Goat anti-Sox17 antibody was purchased from Santa Cruz (Dallas, TX, USA). Alexa Fluor 647 (donkey anti-goat) and Alexa Fluor 488 (donkey anti-mouse) were purchased from Life Technologies (Carlsbad, CA, USA). The live/dead staining kit was obtained from Invitrogen (Waltham, MA, USA). Paraformaldehyde (PFA) was purchased from Election Microscopy Sciences (Hatfield, PA, USA). DAPI was purchased from BD Bioscience (San Jose, CA, USA).

### Fabrication of magnetic microcapsules

Standard soft lithography techniques were used to fabricate PDMS-based droplet microfluidic devices. The stem cell encapsulation process and crosslinking mechanism were performed as described in previous published studies^37,49^. The final dimensions (height) of microfluidic devices were 120 μm for the core channel, 200 μm for the shell channel, and 300 μm for the oil and serpentine channels (Fig. S1). A filter device connected upstream of the encapsulation device was also fabricated and used to improve cell suspension homogeneity in the core microchannel^49^. To produce microcapsules consisting of a liquid core surrounded by a hydrogel shell, four different solutions were loaded into the microfluidic devices: (1) core solution with 8% (w/v) pure PEG and 17% (v/v) Optiprep densifier; (2) shell solution with 4% (w/v) PEG-DAA, 8% (w/v) PEG4MAL, and 15 mM TEA; (3) shielding oil with mineral oil and 0.5% Span-80; and (4) a cross-linking emulsifier with 60 mM DTT dispersed in mineral oil with 3% Span-80. Each solution was infused into microfluidic device at the following flow rates: core (4 μL/min), shell (4 μL/min), shielding oil (20–100 μL/min), and cross-linking oil (60 μL/min). Meanwhile, 1–5 mg/mL magnetic nanoparticles (MNPs) were mixed with the core or shell precursors to fabricate magnetic microcapsules. For clarity, microcapsules without MNPs are denoted as Non-mag microcapsules; microcapsules with MNPs encapsulated in the core are denoted as Mag core; microcapsules with MNPs encapsulated in the shell are denoted as Mag shell. Fabricated microcapsules were retrieved into multi-well plates or bioreactors as described previously^37^.

### Characterization of magnetic microcapsules

To characterize core-shell structure, two different fluorescent labeled nanoparticles were encapsulated in core (150 nm, green) and shell (350 nm, red), respectively. The brightfield and fluorescent images of microcapsules were obtained with an inverted fluorescence microscope (IX83; Olympus, Center Valley, PA, USA) and a confocal microscope (LSM 780; Zeiss, Chicago, IL, USA). Relative fluorescent intensity was analyzed using ImageJ. Additionally, the surface morphology and elemental composition of lyophilized magnetic microcapsules were observed by Field-Emission Scanning Electron Microscopy (FE-SEM; Hitachi S-4700, Hitachi High Technologies America, Inc., Schaumburg, IL, USA). Magnetic microcapsules were first sputter coated with gold-palladium (Au/Pd) to a 5 nm thickness (EMS150R ES, Electron Microscopy Sciences; Hatfield, PA, USA) and then loaded onto a FE-SEM for imaging under 30 kV accelerating voltage. The energy dispersive spectroscopy (EDS) was employed to analyze the contents of carbon (C), oxygen (O), sulfur (S), and iron (Fe) elements in all samples.

### Magnetic responsiveness of microcapsules

To evaluate the magnetic-guided motion, the various magnetic microcapsules were prepared and placed in a PBS-containing glass vial or 12-well plate. Neodymium external magnet (rectangle or disk type) was used to control the movement of the magnetic microcapsules. The magnetic attraction process of the magnetic microcapsules by external magnetic field was recorded using a stereoscope (Stemi 508, Zeiss) equipped with a color camera (Axiocam 305 color, Zeiss).

### Characterizing efficiency of media change in a stirred bioreactor

A solution containing fluorescent dextran was used to assess efficiency of media change in the stirred bioreactor afforded by magnetic microcapsules. Thousand microcapsules were placed in a 5 mL bioreactor containing 10 µM FITC-labeled dextran (70 kDa) dissolved in PBS. After maintaining microcapsules in a stirred bioreactor at 70 rpm with FITC-dextran, the solution was changed by introducing non-fluorescent 1 x PBS. Two scenarios for media change were explored. (1) Microcapsules dispersed in a bioreactor were allowed to settle at the bottom by gravity for 30 min. Afterward, 4 mL of the supernatant was collected in a Falcon tube for analysis, and 4 mL of fresh PBS was added. (2) Magnetic microcapsules dispersed in a stirred bioreactor were guided to one side of the bioreactor by an external magnet. Then, the bioreactor was tilted to the other side, and all supernatant was collected in a Falcon tube for analysis, while 5 mL of fresh PBS was added. This procedure was repeated five times to analyze the dilution FITC-dextran. To quantify residual solute molecules, the solution was analyzed using UV–VIS spectroscopy (NanoDrop Onec, Thermo Scientific Inc., Rockford, IL, USA). The absorption spectrum was recorded, and the relative absorbance intensity at 488 nm was normalized to the initial intensity for the respective sample. The solution was also transferred to a 12-well plate and imaged to quantify fluorescence intensity of the residual FITC-dextran. The average fluorescence intensity was determined and normalized using ImageJ software.

### Culture and encapsulation of hPSCs

HUES-8 is an hESC cell line. These cells were cultured as spheroids in a spinner flask (stirred bioreactor) with mTeSR medium at 70 rpm as described previously^39^. For cell encapsulation experiments, all operations were performed under sterile conditions. HUES-8 cells were dissociated into single cells using an Accutase solution and then re-suspended in the viscous core solution at a concentration of 5 × 10^7^ cells/mL. The prepared cell suspension was passed through a filter device to trap cell clumps before being injected into an encapsulation device as described in published work^49^. The microcapsules carrying HUES-8 cells were collected in a 5 mL bioreactor at a density of 1000 capsules per reactor. The bioreactors were then placed on a stir plate and stirred at 70 rpm inside a humidified incubator at 37 °C with 5% CO_2_. To create unencapsulated spheroids, denoted as bare spheroid, HUES-8 cells were suspended in a bioreactor at a density of 5 × 10^5^ cells/mL. When fabricating magnetic microcapsules carrying HUES-8 cells, the concentration of the MNPs was fixed at 2.5 mg/mL and was premixed with either core or shell precursors.

### Viability and spheroid formation of magnetic microcapsules carrying HUES-8 cells

We followed previously established protocols to assess viability of stem cells after encapsulation^37,49^. Briefly, microcapsules carrying stem cells were first stained with calcein-AM (green fluorescence) and ethidium bromide (red fluorescence) and imaged using an inverted fluorescence microscope. Cell viability was determined by calculating the ratio of live cells (green) to the total number of cells (green and red). The diameter of the HUES-8 spheroids was measured at different time points during culture. To investigate the precise location of encapsulated fluorescent MNPs, all microcapsules were pipetted up and down to break the hydrogel shell and release spheroids. The released spheroids were then fixed with 4% PFA, permeabilized by immersion in 0.1% Triton X-100 for 30 min at 25 °C, and subsequently blocked by immersion in 1% BSA for 1 h at 25 °C. After thorough washing with 1x PBS, the spheroids were incubated with a 1:400 dilution of Alexa Fluor 488-conjugated phalloidin for 1 h to visualize actin. The samples were then washed with PBS and stained with DAPI^50,51^. Finally, the stained spheroids were visualized and imaged using a confocal microscope.

### Pluripotency maintenance and definitive endoderm differentiation of encapsulated HUES-8 spheroids

We characterized pluripotency maintenance and differentiation of HUES-8 cells in three types of microcapsules: Non-mag capsules, Mag core and Mag shell. Bare spheroids (no capsules) represented another experimental group. All groups were cultured in 5 mL stirred bioreactors with mTeSR medium at 70 rpm. The culture medium was replaced as mentioned above: (1) Non-mag capsules and bare spheroids dispersed in the bioreactors were allowed to settle at the bottom by gravity for 30 min. Then, 4 mL of the supernatant was aspirated, and 4 mL of fresh mTesR medium was added. (2) Capsules with Mag core and Mag shell were directed to one side of the bioreactor using an external magnet. Subsequently, the bioreactor was tilted to the opposite side, the total volume (5 mL) of the bioreactor was aspirated and replaced with 5 mL of fresh mTesR media. An established endoderm differentiation protocol with Activin A and CHIR99021 was used to test the effect of the magnetic microcapsule system on the definitive endoderm signals^39,50^. Three types of microcapsules and bare spheroids were exposed to S1 medium supplemented with Activin A and CIR9902 after being maintained in a pluripotent state for 3 days. The medium was changed to S1 medium containing 100 ng/mL activin A after 24 h. All endodermal differentiation experiments lasted for 3 days.

### Assessment of pluripotency and definitive endoderm with RT-PCR and immunofluorescence staining

To assess pluripotency and definitive endoderm gene expression levels, microcapsules containing stem cell spheroids were lysed using an electronic pestle for 3 min. Total RNA was extracted and purified using the RNeasy Plant Mini Kit (Qiagen, Valencia, CA, USA) according to the manufacturer’s instructions^49^. cDNA was synthesized using the QuantiTect Reverse Transcription Kit (Roche, Basel, Switzerland) and quantitative real-time RT-PCR was conducted with the QuantStudio™ 5 System (Thermo Scientific Inc., Rockford, IL, USA) using SYBR green^30,49^. As described in the previous paper, the final analysis was performed based on the threshold cycle using the ΔΔCT method and normalized to glyceraldehyde 3-phosphate dehydrogenase (GAPDH) as a housekeeping gene. The primer sequences used for RT-PCR are listed in Table S1.

For immunofluorescence staining, both encapsulated and unencapsulated spheroids were fixed with 4% PFA and then immersed in a 30% sucrose solution for 24 h. All microcapsules were pipetted repeatedly to mechanically disrupt the hydrogel shell. The spheroids were subsequently embedded in an optimal cutting temperature compound for 1 h, frozen, and sectioned into 10 μm thick slices using a cryostat instrument (Leica CM1950; Leica Biosystems Inc., Buffalo Grove, IL, USA). Next, the sections were permeabilized by immersion in 0.1% Triton X-100 and blocked by immersion in 1% BSA. After thorough washing with PBS, the sectioned spheroids were incubated with 5 μg/mL anti-Sox2 antibodies or 5 μg/mL anti-Sox17 antibodies for 1 h at 25 °C. Following this, the sections were washed with PBS and subsequently incubated with the corresponding secondary antibodies, conjugated to 2 μg/mL Alexa Fluor 488 for Sox2 and 2 μg/mL Alexa Fluor 647 for Sox17, for 1 h at 25 °C in the dark. The samples were then washed with PBS and stained with DAPI^49,52^. Imaging was conducted using a confocal microscope.

### Statistical analysis

At least three tests were conducted to compute the means and standard deviations. A *t*-test was employed to ascertain statistically significant differences, with significance thresholds set at *p < 0.05, **p < 0.01, and ***p < 0.001.

## Results and discussion

**Scheme 1:**
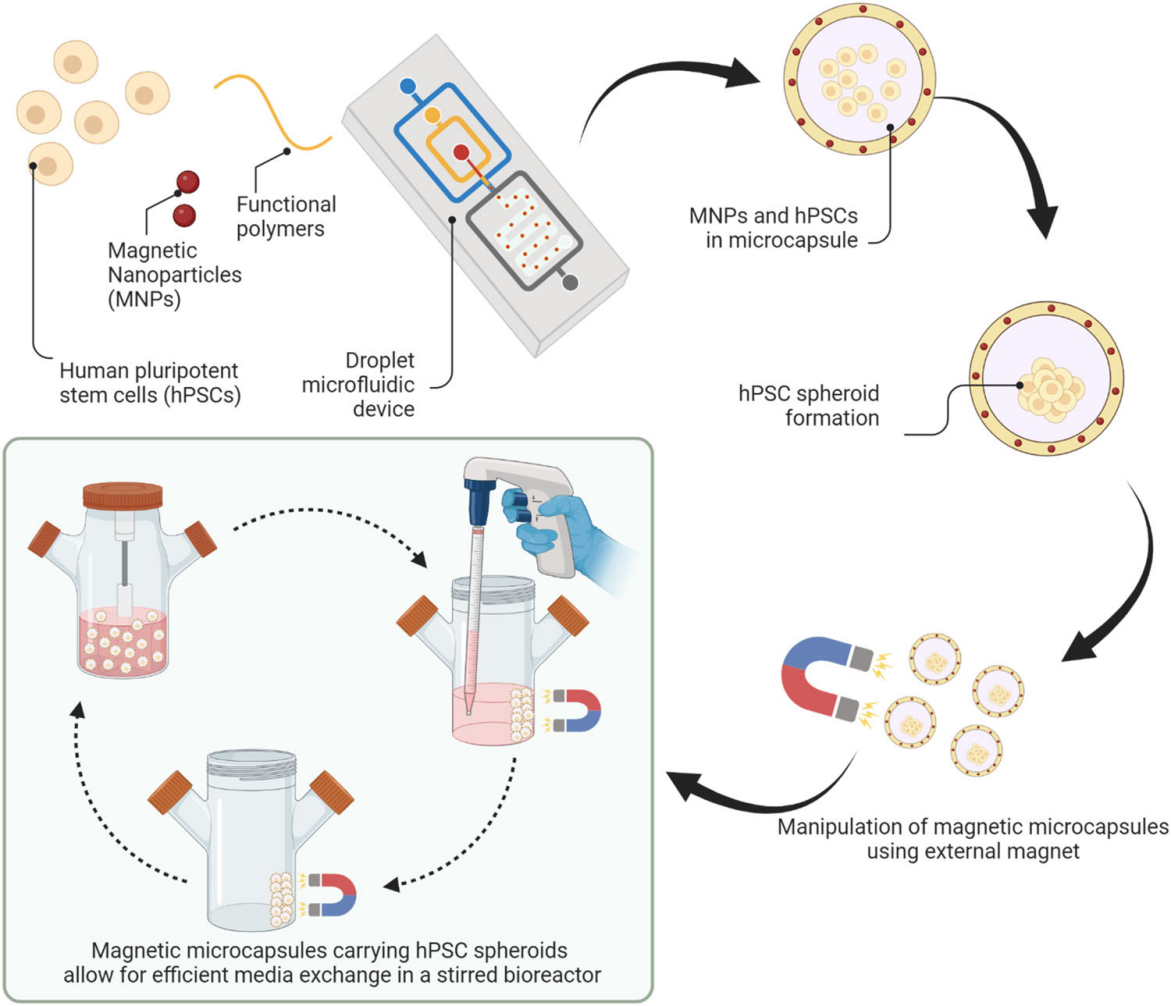
**Schematic that describes fabrication of magnetic microcapsules with entrapped stem cells**. When cultured in a stirred bioreactor, magnetic microcapsules allowed for more efficient media change and produced better stem cell differentiation outcomes

In this study, we wanted to characterize magnetic microcapsules as carriers of hPSC spheroids and focused on assessing microcapsule composition and stem cell phenotype. We also demonstrated the utility of this microcapsule technology for cultivating stem cells in a stirred bioreactor. The design of this study is shown in Scheme 1.

### Fabricating magnetic microcapsules

A co-axial flow-focusing microfluidic device was used to produce magnetic microcapsules carrying stem cells (Fig. S1 and refs. ^37,49^). In this workflow, individual HUES-8 cells were dispersed within a viscous and non-crosslinkable core solution while the shell stream contained the crosslinkable components, PEG-DAA and PEG4MAL. These two aqueous streams were introduced into a co-axial flow-focusing microfluidic device and discretized into droplets at the first oil junction. These droplets contained core-shell structure and were crosslinked at the second oil junction using a di-thiol molecule, DTT. Non-reactive high viscosity components (PEG and Optiprep densifier) leached out from the core and were replaced by water molecules after the microcapsules were transferred into aqueous medium. This resulted in microcapsules with a thin hydrogel shell and an aqueous core. Fluorescently-labeled MNPs were introduced into either the core or the shell stream for incorporation into microcapsules.

### Characterizing microcapsule structure and composition

**Fig. 1:**
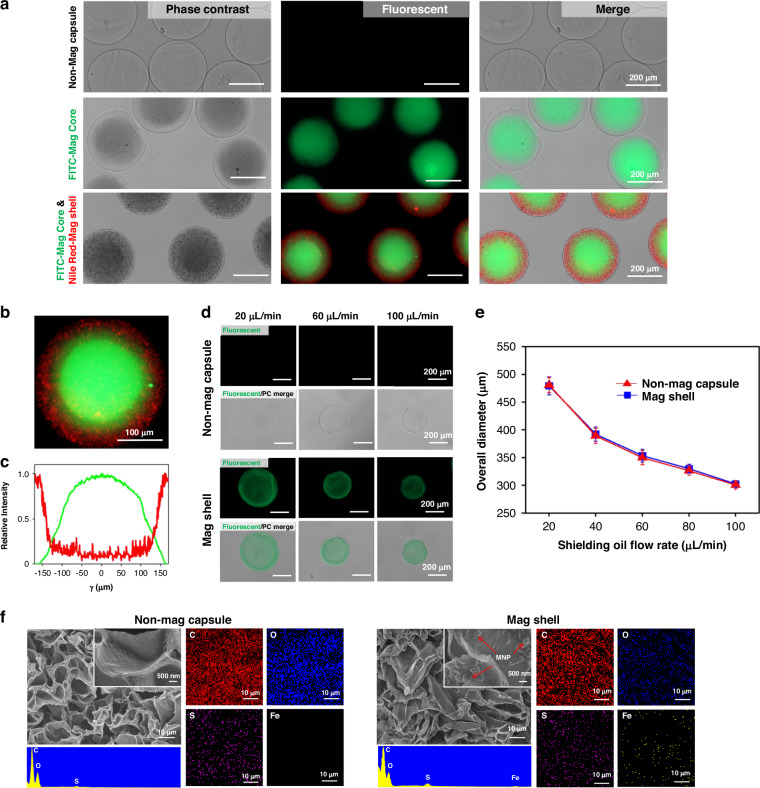
Characterization of magnetic microcapsules. **a** Phase contrast, fluorescent, and merged images of microcapsules (1) without MNPs, (2) with FITC-labeled MNPs in the core only, and (3) with both FITC-labeled MNPs in the core and Nile Red-labeled MNPs in the shell. **b**, **c** Analysis of individual microcapsules carrying red fluorescent and green fluorescence MNPs in the shell and the core respectively. This analysis shows that fluorescence signals are spatially resolved with minimal cross-contamination between the shell and core compartments of microcapsules. **d** Fluorescent and fluorescence/phase contrast merged images of Non-mag capsule and Mag shell at varying shielding oil flow rates. **e** Overall diameter as a function of shielding oil flow rates (core: 4 μL/min, shell: 4 μL/min, shielding oil: 20–100 μL/min, crosslinker oil: 60 μL/min). **f** SEM images of a freeze-dried Non-mag capsule and Mag shell with accompanying EDS spectra and corresponding elemental maps

To confirm the core–shell structure of the magnetic microcapsule, we added fluorescently labeled MNPs to the core (150 nm, green fluorescence) and to the shell streams (350 nm, red fluorescence). Capsule diameter and shell thickness may be controlled by adjusting core-to-shell flow rate ratios, with shell thickness being 15.5 ± 1.5 μm for 1:1 ratio and 40.0 ± 2.5 μm for 1:4 ratio^42^. However, a higher number of cells was trapped in the 40 μm thick shells. Therefore, we chose to operate microfluidic encapsulation device with core and shell flow rates in a 1:1 ratio (4 μL/min), producing microcapsules with ~15 μm hydrogel shell and ~350 μm diameter (Fig. 1a). Close up view of one microcapsule shows a spatially resolved fluorescence profile with clear separation between the green fluorescence in the core and the red fluorescence in the shell (Fig. 1b, c). As shown in Fig. 1d, e, the overall diameter of both Non-mag and Mag capsules could be tuned by adjusting the shielding oil flow rate. Presence of MNPs did not affect tunability of microcapsule dimensions. SEM-EDS was employed to characterize the surface topography and elemental composition of the Non-mag capsule and Mag shell. This analysis confirmed that MNPs were confined to the hydrogel shell of microcapsules (Fig. 1f).

### Manipulating microcapsules in the magnetic field

**Fig. 2:**
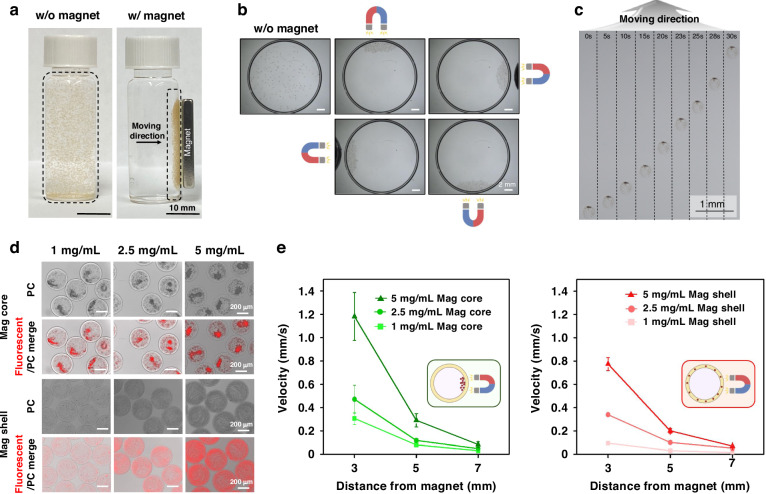
Evaluating magnetic responsiveness of microcapsules. Representative photographs of (**a**) Mag capsules with or without an external magnetic field and (**b**) magnetic-guided rotational motion. (**c**) The movement of a Mag capsule under an external magnetic field; the velocity of the capsule increases with decreasing distance from the magnet. **d** Comparison of Mag core and Mag shell with varying MNP concentrations, demonstrating MNPs aggregation in the aqueous core compared to MNP dispersion when entrapped in the hydrogel shell. **e** Velocity of Mag capsules at various distances from an external magnet as a function of MNP concentration in the core or the shell. The results indicate that external that velocity toward the source of magnetic field increased with higher MNP concentration, and that Mag core traveled faster than Mag shell capsules for the same MNP concentration

We investigated the movement of Mag capsules (Mag core and Mag shell) under an external magnetic field. Figure 2a shows Mag capsules that are well-dispersed in the absence of magnet and then concentrated adjacent to the magnet. The direction of movement of the Mag capsules could also be controlled by adjusting the position of the magnetic field, as illustrated in Fig. 2b. Movement of microcapsules in the magnetic field was quantified using brightfield microscopy. As seen from representative time-lapse images in Fig. 2c, a magnetic capsule moved toward the source of the magnetic field with increasing velocity with increased proximity to the magnet.

Magnetic properties of microcapsules depend on the amount of encapsulated MNPs. To evaluate magnetic responsiveness, we encapsulated various concentrations of MNPs in either the core or the shell using same core and shell flow rates (4 µL/min core and 4 µL/min shell, respectively). Therefore, we hypothesized that the same amount of MNPs could potentially be encapsulated either in the core or the shell. However, microcapsules with MNPs in the core (Mag core) or shell (Mag shell) behaved differently. MNPs in the core were initially well-dispersed in the viscous core solution. However, as the viscous contents of the core diffused out over time and were replaced by aqueous media, MNPs aggregated in response to gravity and entropy. In contrast, MNPs remained stably trapped and dispersed within the hydrogel network of the shell (see Fig. S2).

Mag capsules containing three different concentrations of MNPs (1, 2.5, and 5 mg/mL) in either the core or shell are depicted in Fig. 2d. The attraction force (${\vec{F}}_{{mag}}$) exerted on a microcapsule for a given MNP density, creates magnetization $\vec{m}$, with magnetic field $\vec{B}$ as given by the formula:

$${\vec{F}}_{{mag}}=\left(\vec{m}\,\cdot\, \nabla \right)\,\vec{B}\,$$

The strength of the dipole field decays cubically with distance^53^, therefore microcapsules move towards the magnetic field with increasing velocity. We evaluated the velocity of the Mag capsules at distances of 3, 5, and 7 mm from the magnet. By altering MNP density, and hence, ${\rm{m}}$, at the 3 mm distance, the Mag core velocity increased from 0.31 ± 0.05 mm/s at an MNP concentration of 1 mg/mL to 1.18 ± 0.21 mm/s at an MNP concentration of 5 mg/mL. Similarly, at the same distance of 3 mm, the Mag shell capsules increased velocity from 0.09 ± 0.01 mm/s for 1 mg/mL MNPs to 0.77 ± 0.06 mm/s for 5 mg/mL MNPs.

Interestingly, our analysis revealed that Mag core moved faster than Mag shell (Fig. 2e). This result is likely due to magnetic anisotropy created by the self-assembly of superparamagnetic nanoparticles free to move within the core when exposed to a global external field. Self-assembly formed an easy axis that follows the direction of the magnetic field as described before^54^. The easy axis results in higher local magnetization compared to randomly distributed particles. Similar effect was employed previously for locomotion of magnetic microswimmers based on superparamagnetic particles^55^. However, nanoparticles fixed within the gel matrix of the shell were unable to align/assemble, so that no easy axis could be formed to increase the magnetic pulling force on the microspheres (Fig. 2e). Based on this characterization, we chose to use the highest amount of magnetized microcapsules (5 mg/mL MNPs) for subsequent hPSC encapsulation and cultivation experiments. Overall, we demonstrated that microcapsules with either a Mag core or Mag shell may be manipulated in the external magnetic field.

### Using magnetic microcapsules to improve efficiency of media change in a stirred bioreactor

**Fig. 3:**
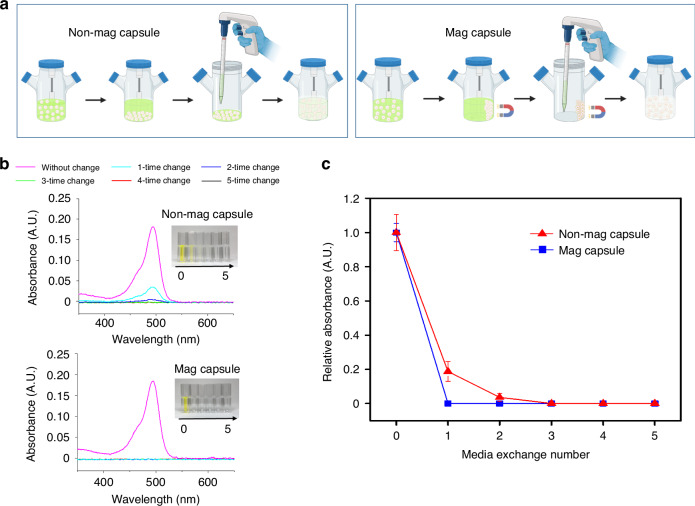
Evaluating efficiency of media change in a stirred bioreactor. **a** Schematic illustration of the media replacement methods being compared. Non-mag capsules and Mag capsules were maintained in a 5 mL stirred bioreactors containing solution of FITC-dextran. Media was changed by pausing agitation until capsules or spheroids settled down and 80% of solution was removed by aspiration. In the case of Mag capsules, an external magnet was applied to hold capsules in place to allow removal of ~100% of solution. **b** UV–Vis analysis of residual FITC-dextran following solution replacement. **c** Relative absorbance at 488 nm normalized by the initial absorbance in solution in the bioreactor

Solution containing FITC-dextran was used to characterize efficiency of media change afforded by Mag microcapsules. When working with Non-mag capsules or bare spheroids, we followed a protocol typically used for changing media in stirred suspension cultures (Fig. 3a). The stirring was stopped, and spheroids or capsules were allowed to sediment for 30 min. 80% of solution (4 mL) was then removed and replaced with non-fluorescent 1 × PBS. This is standard procedure for suspension cultures designed to minimize loss of spheroids or capsules during media change. In the case of Mag capsules, an external magnet was used to keep capsules in place while ~100% of the solution was replaced. Figure 3b, c quantifies residual FITC-dextran as a function of media change cycles. These results show that the protocol utilizing Mag capsules allowed for more efficient solution change.

### Characterizing viability and proliferation of hPSC spheroids in magnetic microcapsules

**Fig. 4:**
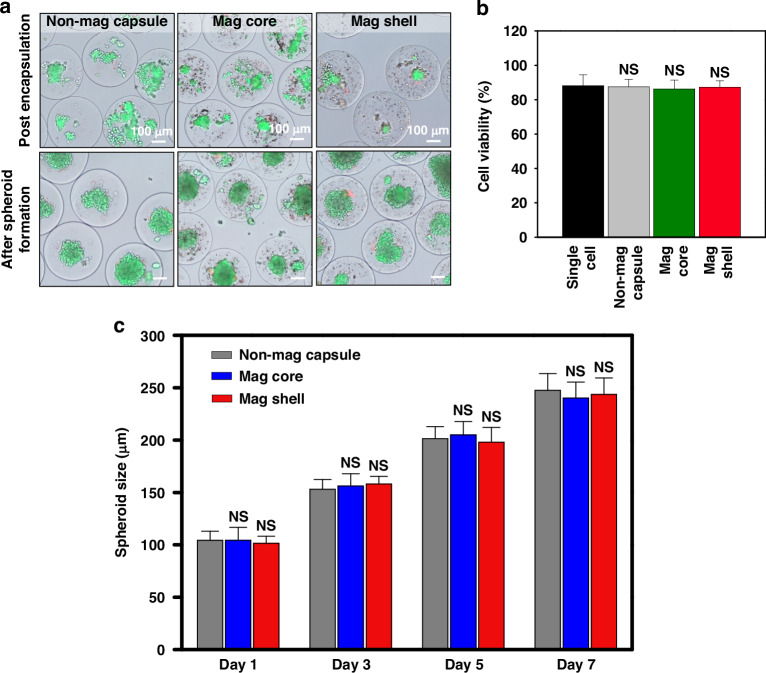
Viability and proliferation of hPSC spheroids in magnetic microcapsules. **a** Live/dead fluorescence images at day 0 and day 3 and (**b**) quantitation of viability for hPSCs in microcapsules (Non-mag capsule, Mag core and Mag shell) as well as single hPSCs before encapsulation. **c** Characterizing change in stem cell spheroid diameter over time revealed no differences regardless of presence and location of MNPs (NS: not significant). ‘Non-mag capsule’ describes stem cell spheroids in microcapsules without MNPs, ‘Mag core’ describes a condition with MNPs in the core of a microcapsule, in direct contact with stem cells. ‘Mag shell’ refers to MNPs trapped in the hydrogel shell and not in direct contact with stem cells. Data from (**b**, **c**) are presented as mean ± standard deviation and statistically evaluated by Student’s *t*-test. Each group was compared to single cell for (**b**) and Non-mag capsule for (**c**), with the minimum level of significance set at *p* < 0.05 (NS: not significant, *p < 0.05, **p < 0.01, and ***p < 0.001)

As the next step, we investigated whether incorporating MNPs into the microcapsules affected the viability and proliferation capacity of HUES-8 cells—a human embryonic stem cell (hESC) cell line. These cells are referred to as either HUES-8 or hPSCs throughout the paper. We used live/dead staining to assess viability immediately after encapsulation. As depicted in Fig. 4a, b, the cell viability of the single cell, Non-mag capsule, Mag core, and Mag shell was 88.2 ± 6.3%, 87.5 ± 4.3% and 86.2 ± 5.3% 87.3 ± 3.7%, respectively. We also performed L/D staining after spheroid formation at day 3, showing high viability for spheroids in all capsules (Fig. 4a). Thus, the initial cell viability was not affected by the incorporation of MNPs with the cells.

We characterized the effects of MNP incorporation on stem cell proliferation. Encapsulated hPSC spheroids were cultured in pluripotency maintenance mTeSR medium for a total of 7 days and were imaged to assess changes in spheroid diameter. Fig. S3 shows representative images of encapsulated HUES-8 spheroids on day 1, day 3, day 5, and day 7. For the Non-mag capsules, single spheroids were formed inside the microcapsules. For the Mag core condition, a complex spheroid consisting of both MNPs and HUES-8 cells was formed inside the microcapsule. For Mag shell, HUES-8 spheroids formed in the core region while MNPs were evenly distributed in the hydrogel shell. As shown in Fig. 4c, S3, spheroid formation and growth were similar in all experimental groups. The results in this section indicate that presence of MNPs, whether in the core or shell, had no discernible effect on the initial viability, spheroid formation, or proliferation of HUES-8 cells. In addition, we characterized distribution of MNPs inside microcapsules using fluorescence microscopy. As shown in Fig. S4a, fluorescent MNPs in the Mag core capsules were incorporated into and observed at different focal planes within stem cell spheroids. The presence of MNPs did not interfere with spheroid formation and structure (Fig. S4b).

### Assessing pluripotency of encapsulated hPSC spheroids

**Fig. 5:**
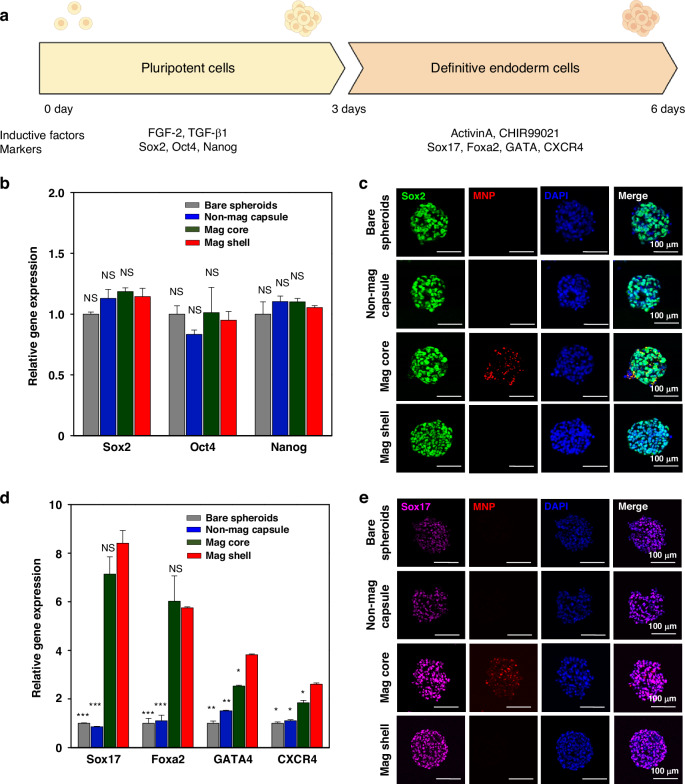
Pluripotency and definitive endoderm expression in magnetic microcapsules. **a** Workflow for differentiating encapsulated HUES-8 cells into definitive endoderm (DE). **b** Pluripotency gene expression (Sox2, Oct4, and Nanog) assessed by RT-PCR. Experimental groups: bare spheroids and three types of microcapsules (Non-mag capsule, Mag core, and Mag shell). **c** Immunofluorescent staining for Sox2 expression for unencapsulated and encapsulated HUES-8 spheroids. **d** Expression of DE markers analyzed by RT-PCR. **e** Immunofluorescence staining for Sox17. Data from (**b**) and (**d**) are presented as mean ± standard deviation and statistically evaluated by Student’s *t*-test. Each group was compared to Mag shell, with the minimum level of significance set at *p* < 0.05 (NS: not significant, *p < 0.05, **p < 0.01, and ***p < 0.001)

The pluripotency of HUES-8 was evaluated by RT-PCR and immunofluorescence staining^49,56^. As described in Fig. 5a, encapsulated hPSCs were maintained under pluripotency-inducing conditions for 3 days. Four experimental groups were tested: (1) bare spheroids, (2) Non-mag capsule, (3) Mag core, and (4) Mag shell. Stem cells in all experimental groups were cultured in mTeSR media containing pluripotency-inducing factors with FGF-2 and TGF-β1. However, for bare spheroids and Non-mag capsules, 80% of the culture medium was replaced with fresh media. In the case of Mag core and Mag shell, ~100% of the culture medium was replaced with fresh media, as described above. RT-PCR analysis for markers of pluripotency (e.g., Sox2, Oct4, and Nanog) showed that Mag capsules (Mag core and Mag shell) demonstrated similar levels of pluripotency gene expression as what was observed for bare spheroids and Non-mag capsules. We note that pluripotency gene expression was similar for all groups despite the fact that bare spheroid and non-mag capsule groups were cultured with 80% media change while Mag capsules had ~100% media change. This is not surprising because, in this case, spent and fresh media were of similar compositions. These results are significant, however, because they demonstrate that stem cells co-encapsulated with MNPs either in the core or shell of microcapsules had similarly high pluripotency gene expression compared to bare spheroids and Non-mag capsules—experimental groups without MNPs. The RT-PCR analysis was corroborated by immunofluorescence staining which showed similarly strong expression of the pluripotency marker Sox2 for all experimental groups regardless of presence of MNPs (Fig. 5b, c).

### Endodermal differentiation of HUES-8 spheroids in magnetic microcapsules

While pluripotent stem cells can give rise to adult cell types originating from all three germ layers, we are particularly interested in pancreatic and hepatic cells that originate from endoderm^39,52,57^. In terms of culture method priming for endoderm, we find that endodermal differentiation happens when concentration and duration of endoderm-inducing signals, Activin A and Wnt3A inhibitor, are well-defined. When these signals are suboptimal, efficiency of endodermal differentiation decreases. Therefore, we wanted to evaluate endodermal differentiation of encapsulated HUES-8 spheroids. As seen from Fig. 5d, RT-PCR analysis revealed that both Mag core and Mag shell microcapsules had several-fold higher levels of definitive endoderm gene expression as compared to bare spheroids and Non-mag capsules. Stem cells in Mag shell capsules had modestly, but statistically significant, higher levels of Sox17, GATA4, and CXCD4 gene expression compared to Mag core capsules. Immunofluorescence staining for Sox17 corroborated the RT-PCR results, confirming higher levels and frequency of expression of this endodermal marker in HUES-8 cells within Mag core and Mag shell compared to bare spheroids and Non-mag capsules (Fig. 5e). While, as described in the preceding section, more efficient change of media afforded by magnetic microcapsules did not enhance pluripotency maintenance, it was clearly important when moving from pluripotency (mTeSR) to endoderm-inducing media (with Activin A and CHIR99021). Less efficient differentiation of bare spheroid and Non-mag capsule experimental groups should be attributed to incomplete media change and residual pluripotency signals (e.g. bFGF and TGF-β1) that interfere with the differentiation step (induction of endoderm). In summary, results shown in Fig. 5d, e demonstrate that magnetic microcapsules afford more efficient media change in a stirred bioreactor, resulting in improved endodermal differentiation of hPSCs.

## Conclusion

In summary, we fabricated and characterized magnetic microcapsules carrying hPSC. These microcapsules were comprised of a hydrogel shell and aqueous core and were designed to promote aggregation of stem cells into spheroids. Microcapsules were made magnetic by incorporating MNPs into either the core or the shell. Importantly, the presence of MNPs did not interfere with viability, spheroid formation, proliferation or pluripotency maintenance of hPSCs. To highlight one application of this technology, we demonstrated the benefits of magnetic microcapsules for culturing stem cells in a stirred bioreactor. These microcapsules afforded more efficient media change and resulted in better differentiation outcomes compared to bare spheroids. In the future, we envision using magnetic microcapsules carrying hPSC spheroids to improve scalability and differentiation outcomes for such important adult cell types as pancreatic islets and hepatocytes. Another future application may involve the use of magnetic microcapsules for delivery/transplantation of cellular therapies.

## Supplementary information

Supplementary Information
