# Supplementary material for: Designing magnetic microcapsules for cultivation and differentiation of stem cell spheroids
Source: Microsyst Nanoeng. 2024 Sep 12;10:127. doi: 10.1038/s41378-024-00747-9 (PMC11390961; doi:10.1038/s41378-024-00747-9)
Supplement: Supplementary file 1 — Supplementary Information [file 41378_2024_747_MOESM1_ESM.docx]

**Supplementary Information**

**Designing Magnetic Microcapsules for Cultivation and Differentiation of Stem Cell Spheroids**

Kihak Gwon^1, 4*^, Ether Dharmesh^2^, Kianna M. Nguyen^1^, Anna Marie R. Schornack^1^, José M. de Hoyos-Vega^1^, Hakan Ceylan^3^, Gulnaz Stybayeva^1^, Quinn P. Peterson^1^, and Alexander Revzin^1*^

^1^ Department of Physiology and Biomedical Engineering, Mayo Clinic, Rochester, MN, USA

^2^ Department of Biomedical Engineering, Saint Louis University, St. Louis, MO, USA

^3^ Department of Physiology and Biomedical Engineering, Mayo Clinic, Scottsdale, AZ, USA

^4^ Department of Biofibers and Materials Science, Kyungpook National University,

Daegu, Republic of Korea

*Corresponding authors: [revzin.alexander@mayo.edu](mailto:revzin.alexander@mayo.edu); [khgwon@knu.ac.kr](mailto:gwon.kihak@mayo.edu)


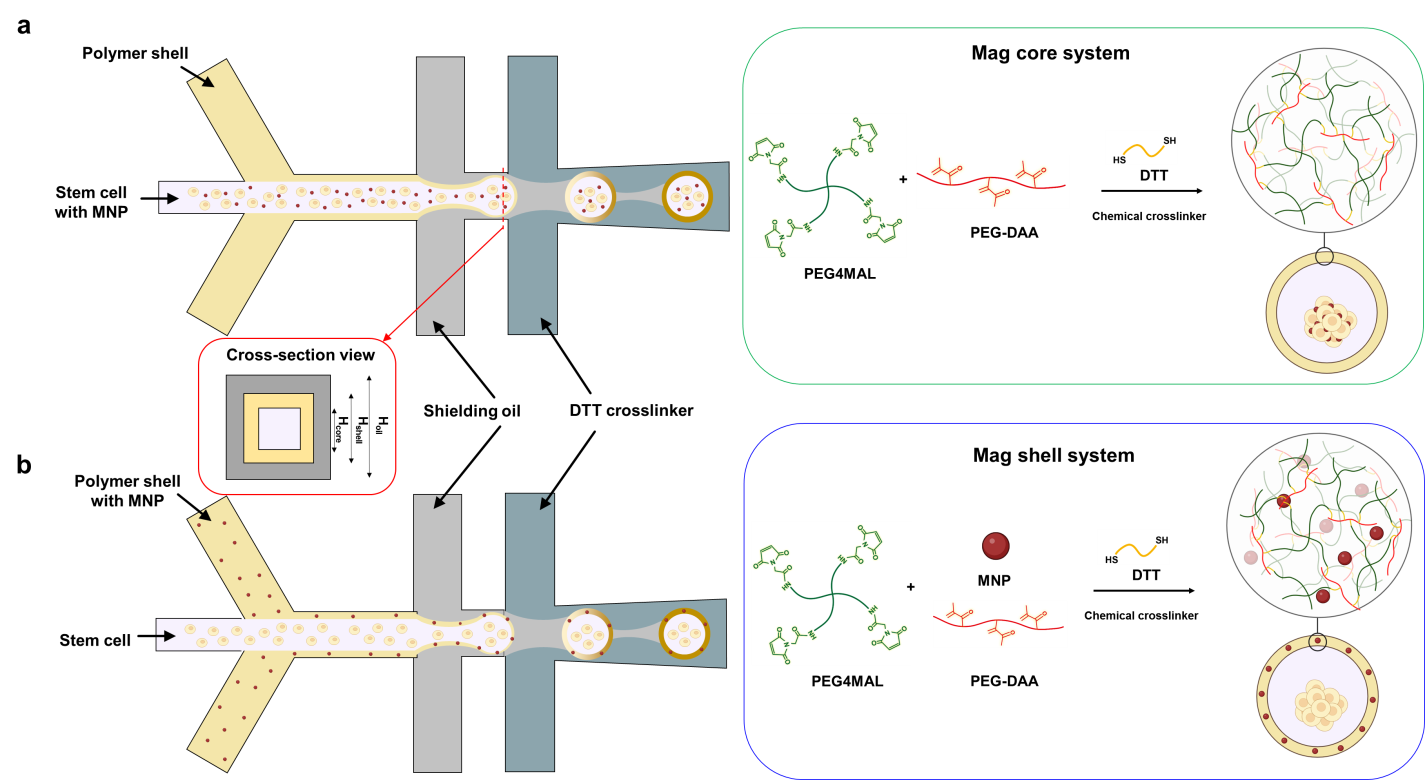


**Fig. S1 Co-axial flow-focusing microfluidic used to fabricate magnetic microcapsules with stem cells**. This device consisted of four inlets (core, shell, shielding oil, and crosslinker oil) and a serpentine channel leading to a droplet collection port. The heights of the core, shell, and oil channels are 120 μm (H_core_), 200 μm (H_shell_), and 300 μm (H_oil_), respectively. (a) HUES-8 cells and magnetic nanoparticles (MNPs) were premixed with a viscous core solution, while the shell stream contains PEG-DAA and PEG4MAL. (b) In an alternative process, HUES-8 cells were dispersed in a viscous core solution, while the shell stream contained PEG-DAA and PEG4MAL with MNPs. These two aqueous streams were introduced into a co-axial flow-focusing microfluidic device, where aqueous droplets formed by mixing with a shielding oil, resulting in the creation of core-shell microcapsules. Subsequently, these microcapsules underwent chemical reaction with DTT crosslinker in the oil phase. Microcapsules with MNPs in the core were denoted ‘Mag core,' with MNPs in the shell - 'Mag shell.'


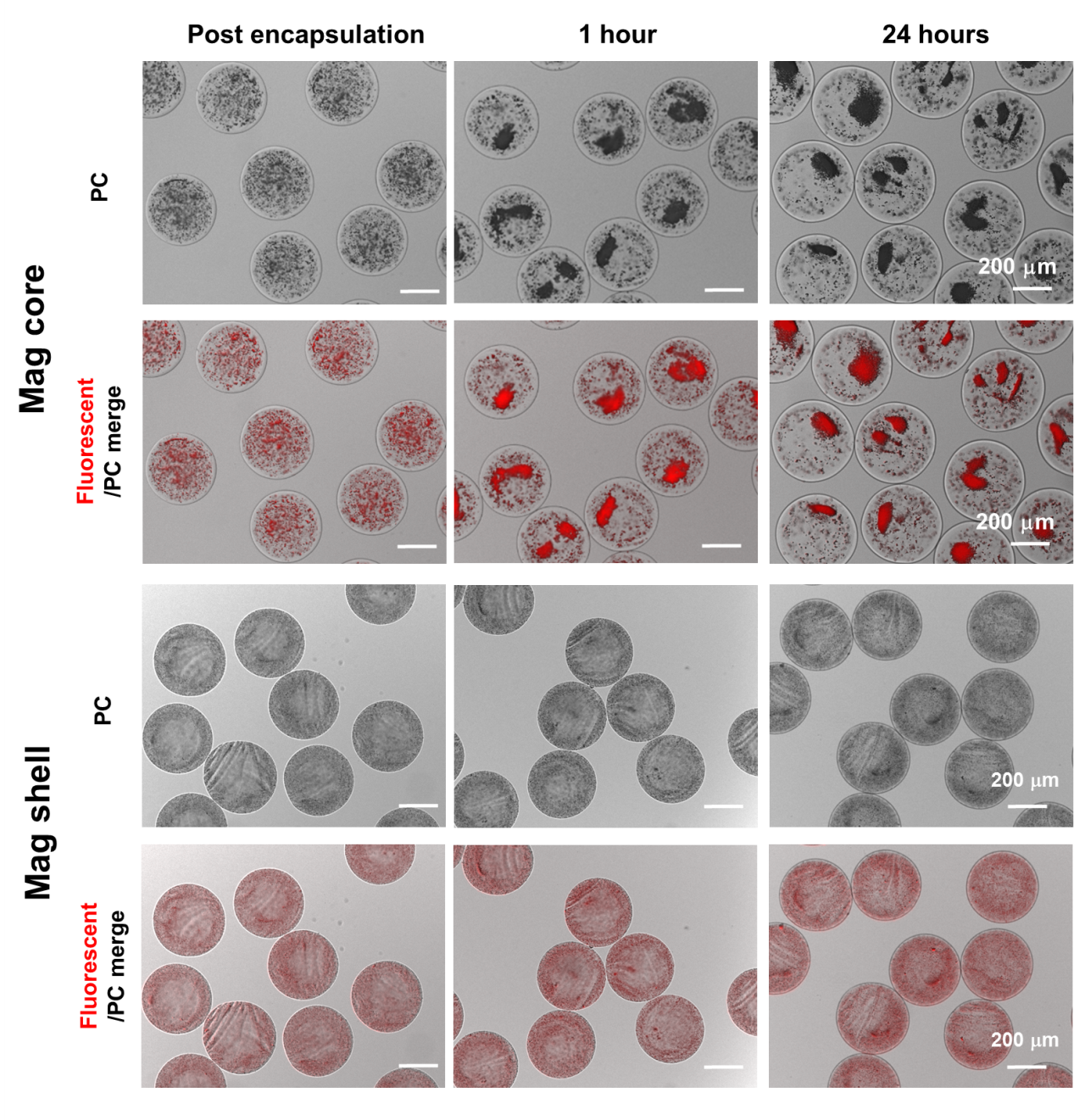


**Fig. S2** **Observations of MNPs entrapped in the core or the shell of microcapsules.** MNPs in the aqueous core were free to move and collected into aggregates due to gravity while MNPs in the shell remained immobilized in the hydrogel network over time.

**
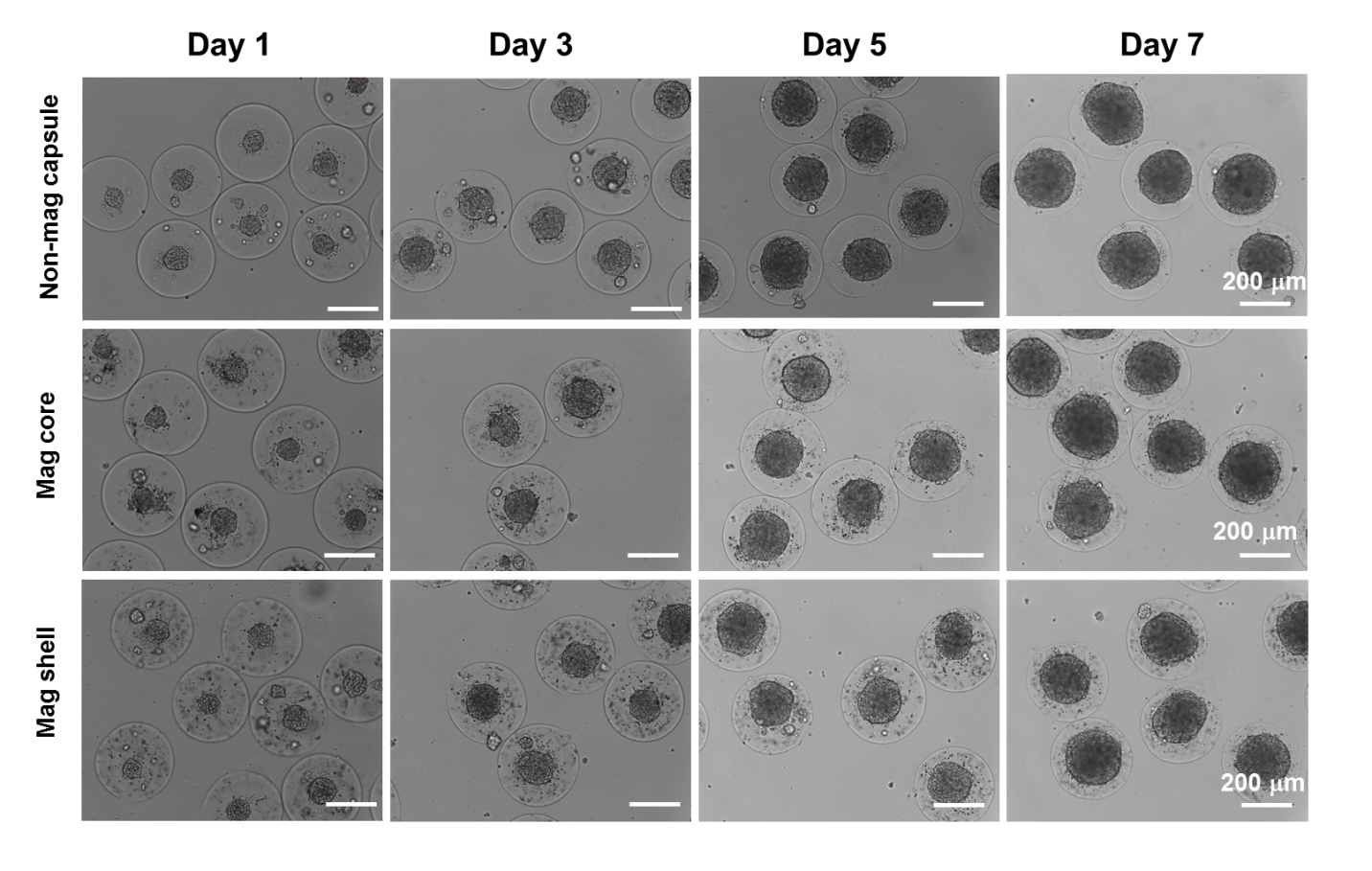
**

**Fig. S3 Representative images of encapsulated hPSC (HUES-8) spheroids cultured in pluripotency inducing media (mTeSR).** These images show that spheroids proliferated at a similar rate regardless of presence of MNPs.


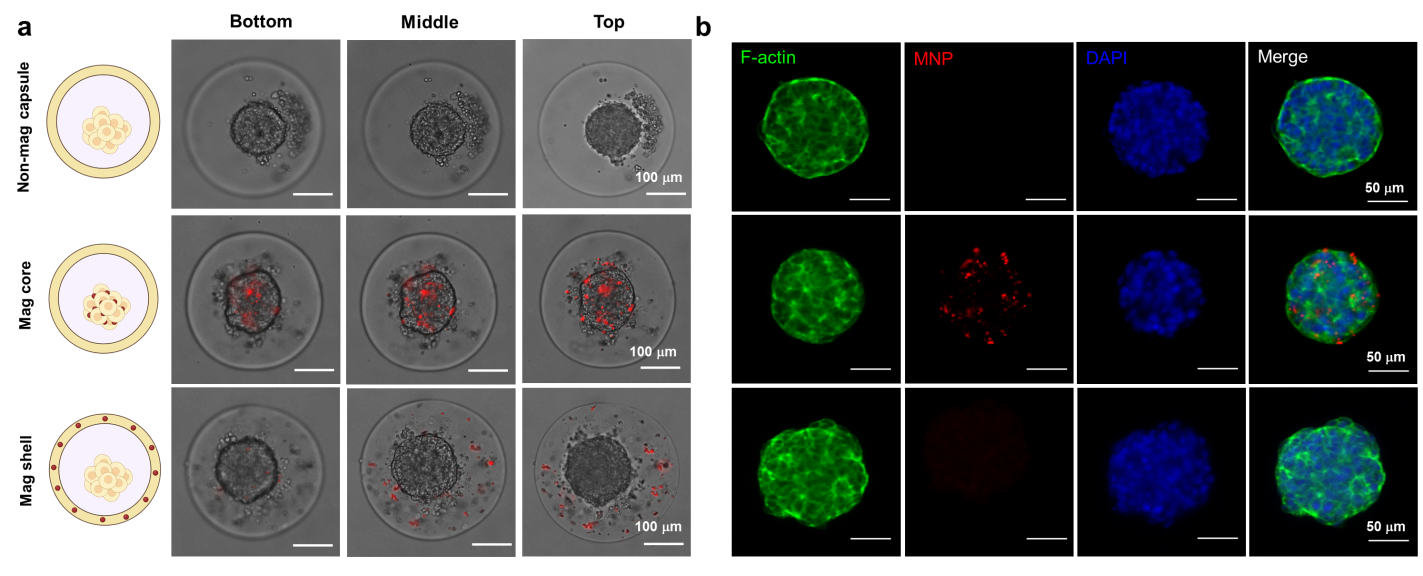


**Fig. S4 Fluorescence and confocal microscope images of hPSC (HUES-8) spheroids in magnetic microcapsules.** (a) Brightfield and fluorescent merged images of the bottom, middle, and top focal planes of microcapsules. Red fluorescence is from Nile Red-labeled MNPs. (b) Confocal microscopy images of HUES-8 spheroids, which were liberated from and analyzed by fluorescence staining. Green fluorescence - F-actin, blue - DAPI and red - Nile Red-labeled MNPs.

**Table S1.** Sequences of primers used in qRT-PCR analysis.

| **Gene** | **Forward** | **Reverse** |
| --- | --- | --- |
| **GAPDH** | **TGTTGCCATCAATGACCCCTT** | **CTCCACGACGTACTCAGCG** |
| **Sox2** | **TCAGGAGTTGTCAAGGCAGAGAAG** | **GCCGCCGCCGATATTGTTATTAT** |
| **Oct4** | **GATCACCCTGGGATATACAC** | **GCTTTGCATATCTCCTGAAG** |
| **Nanog** | **CCGGTCAAGAAACAGAAGACCAGA** | **CCATTGCTATTCTTCGGCCAGTTG** |
| **Sox17** | **GGCGCAGCAGAATCCAGA** | **CCACGACTTGCCCAGCAT** |
| **Foxa2** | **CGAGTTAAAGTATGCTGGG** | **CATGTACGTGTTCATGCC** |
| **GATA4** | **CATCAAGACGGAGCCTGGCC** | **TGACTGTCGGCCAAGACCAG** |
| **CXCR4** | **CTTCATCTTTGCCAACGTCAG** | **GGACAGGATGACAATACCAGG** |
